# Quality assessment of selected co-trimoxazole suspension brands marketed in Nairobi County, Kenya

**DOI:** 10.1371/journal.pone.0257625

**Published:** 2021-09-22

**Authors:** Beatrice Njeri Irungu, Lilian C. Koech, Joyce M. Ondicho, Lucia K. Keter

**Affiliations:** Kenya Medical Research Institute, Centre for Traditional Medicine and Drug Research, Nairobi, Kenya; US Department of Agriculture, UNITED STATES

## Abstract

**Introduction:**

Quality of medicines in both developed and developing countries is sometimes compromised due to infiltration of counterfeit, substandard or degraded medicines into the markets. It is a public health concern as poor quality medicines endanger public health where patients are exposed to chemical toxins and/or sub-therapeutic doses. This could lead to reduced treatment efficacy and promote development of drug resistance. Co-trimoxazole, a fixed dose combination of sulfamethoxazole and trimethoprim, is a broad spectrum for bacterial diseases and is also used as a prophylaxis for opportunistic infections in HIV infected individuals. This study evaluated quality of selected co-trimoxazole suspension brands marketed in Nairobi County, Kenya.

**Methods:**

A total of 106 samples were collected, categorized into 15 brands and evaluated for active pharmaceutical ingredient content (API) and pH following United States Pharmacopeia. Assay for API was conducted using High Performance Liquid Chromatography. Results were compared with pharmacopeia references. Visual examination of labels and confirmation of retention status of the brands with Pharmacy and Poisons Board retention register was carried out.

**Results:**

The samples were primarily of local origin (86.7%). On October 23, 2019, retention status of six of the fifteen brands documented were no longer listed in the Pharmacy and Poisons Board retention register. Of the 106 samples tested 70.6% and 86.8% were compliant with United States Pharmacopeia (USP) specifications for pH and API respectively while 84.0% adhered to packaging and labelling requirements.

**Conclusion:**

This study has demonstrated that majority of co-trimoxazole suspensions tested were compliant with USP requirements. Additionally, it has provided evidence of poor quality co-trimoxazole medicines that could compromise treatment of infectious diseases in children. This emphasizes the need for regular quality assurance tests to ensure only quality medicines are in the market.

## Introduction

Antibiotics such as co-trimoxazole are medicines used for treatment of bacterial infections and microbe-borne diseases. The quality of medicines especially antibiotics is a major global concern due to their high demand [1, 2]. For instance, in 2015 WHO reported overall antibiotics consumption in 65 countries to range from 4.4 to 64.4 defined daily doses per 1,000 inhabitants per day. This measure can be roughly interpreted as the number of individuals per 1000 inhabitants on antibiotics per day with moderate to severe infections [3]. Poor quality antibiotics (substandard, counterfeit or degraded) partially exacerbate antimicrobial resistance leading to an increased cost of treatment and prolonged hospital stays [4].

Existing data suggest a high prevalence of poor quality drugs in South East Asia and Africa [5–10]. In Papua Guinea 35 out of 360 tablets/capsules of different antibiotics analysed had less active pharmaceutical ingredient (API) than pharmacopeia reference ranges. In Ghana, a study of 68 different medicines essential for children reported that 61.8% failed to meet pharmacopeia standards [11], while in Indonesia a study on 104 different antibiotics reported that 18% failed to meet British pharmacopeia standards [12]. There are several quality assessments reports in Kenya that mainly focused on antimalarial drugs [13, 14]. A recent study carried out in Embu County by Ndwigah and Co-workers [2018] [15] evaluated 39 samples of artemisinin based combination tablets and oral suspensions where all passed quality control tests.

There has also been a growing global concern on the quality of antibiotics with several studies reporting antibiotics that failed to meet pharmacopeia requirements [16–19]. Fadeyi and Co-workers [20] reported on quality of antibiotics from Ghana, Nigeria and United Kingdom where 9 out of 15 samples of co-trimoxazole tablets tested failed to meet United States Pharmacopeia limits (USP) while all 20 samples of amoxicillin passed. Koech et al. [2020] [21] reported that of 53 samples of amoxicillin tested, an overall failure of 30.2% was observed.

Co-trimoxazole is a fixed dose combination of two antimicrobial drugs, trimethoprim and sulfamethoxazole. It was introduced in late 1960’s as a broad spectrum for bacterial diseases such as urinary tract infections, respiratory infections, sexually transmitted diseases, gram-negative sepsis, enteric infections and typhoid fever [22]. It is also used as a prophylaxis for opportunistic infections in HIV infected individuals where it has been shown to reduce morbidity and mortality [23, 24]. A number of studies have reported on poor quality co-trimoxazole formulations [25, 26]. In Cambodia a study on quality of selected medicines reported that of 82 samples of co-trimoxazole tablets tested, 9.8% and 18.3% failed to meet USP tolerance limits for content and dissolution, respectively. Similarly, in Malawi, Khuluza [2014] [4] reported that 5 out of 11 co-trimoxazole tablets sampled failed to meet British Pharmacopeia content limits. In Kenya, Abuga *et al*., [2013] [16] reported co-trimoxazole as being one of the antibiotics that failed to meet pharmacopeia requirements.

The primary objective of this study was to determine the quality (content of API, and pH) of selected brands of co-trimoxazole suspension marketed in Nairobi County, Kenya. Secondary objectives were to determine the number of documented brands that were (a) on Kenya Pharmacy and Poisons Board retention register (b) of local origin or imported. Labelling and packaging standard were also visually inspected.

## Materials and methods

### Study location

This study was undertaken in Nairobi County, the capital city of Kenya and the gateway for imports and exports thus the commercial hub of the country. In addition, it is the headquarters of majority of the pharmaceutical distributors and wholesalers in the country [21, 27, 28]. Nairobi has seventeen Sub-counties which are further divided into eighty-five wards. It has a population of about 4.3 million, contributing to 9.2% of the Kenyan population [29].

### Reference materials

Primary reference standards of trimethoprim and sulfamethoxazole (≥ 99%) were obtained from United States Pharmacopeia manufactured in India and working standards (>99%) were from Andhra Organics limited. Solvents were of HPLC grade, methanol (Merck, Germany), acetonitrile (Merck, Germany) while triethylamine (FINAR, India) and sodium hydroxide salt (Merck, Germany) were of analytical grade.

### Sample collection

According to Pharmacy and Poisons Board- Kenya register as of 31^st^ December2015, there were 1,374 registered pharmacy premises in Nairobi County (www.Pharmacyboardkenya.org/?p=530). In the 17 sub-counties, 39 wards were selected based on economic stratification; low, middle and high income classes [30, 31]. The Pharmacy premises list from the Kenya Poisons and Pharmacy Board did not indicate pharmacy location. Hence, 309 pharmacies were purposively sampled for drug purchase using Krejcie and Morgan sample table [32]. A total of 224 oral suspensions were sampled from retail pharmacies between May and June of 2019. To eliminate bias, samples of all the co-trimoxazole brands stocked in each pharmacy sampled were purchased. For each brand two bottles of the same batch numbers were purchased. Samples from the same manufacturer but of different strength and /or batch number were classified as an individual sample. Each sample was accorded a unique code based on location.

Secondary sampling was then undertaken, where samples from the same ward with similar batch numbers were removed resulting to 188 oral suspensions. Systematic sampling was finally done to achieve our target sample size of approximately 100 oral suspensions. A random starting point and a fixed periodic interval was selected as shown below [33].


$$periodic\;interval=initial\;sample\frac{size}{target}sample\;size=\;\frac{188}{100}=1.88\;\approx 2$$

In addition, priority was given to unique brands and samples that had any labelling and packaging inconsistencies after visual inspection, this resulted to a sample size of 106, which were analysed. The samples were stored at Kenya Medical Research Institute, Centre for Traditional Medicine and Drug Research, pharmaceutical laboratory away from light, below 30°C as per manufacturers’ requirement.

### Determination of API content by HPLC

The determination of sulfamethoxazole and trimethoprim potency was done according to criteria established by the United States Pharmacopeia (USP) [34]. The HPLC analyses was done on an Agilent 1260 Infinity series (Agilent Technologies, Deutschland, Germany) supported by Open-Lab software version A.01.03. A stainless steel LiChrospher^®^ 100 RP-18 end capped column (30cm × 3.9mm) was packed with Octadecylsilyl silica gel. The injection volume was maintained at 10μL, flow rate at 1.5ml/min and wavelength at 254nm. Column temperature was maintained at 40°C. The percentage label claim (% L.C.) of each drug sample was obtained by comparing the average peak areas and concentrations of both the standard and sample solutions taking into account the volume taken, the purity of the standard and the label claim contents of each sample.

### Method system suitability parameters

System suitability test was done routinely before sample analysis to verify resolution, accuracy and repeatability of chromatographic tests. In addition, each sample was analysed in triplicates and each injected thrice. Predefined acceptance criteria for API analysis used in this study are as shown in Table 1.

**Table 1:** Method suitability parameters.

Parameter	Method system suitability Parameters		Acceptable Criteria
	Sulfamethoxazole	Trimethoprim	
**Precision /Injection Repeatability**	Relative Standard Deviation indicated in the data for respective samples in the results tables		Relative Standard Deviation ≤2%
**Resolution Factor (R)**	2.01 to 2.96		R> 1.5
**Tailing Factor (T)**	0.76 to 1.32	0.87 to 1.7	T≤ 2
**Theoretical Plates (N)**	2233–3556	2970–4589	N≥ 2000 Plates

### Determination of pH of co-trimoxazole suspensions

The pH of each sample was determined with a Thermo scientific pH meter (Orion VERSA STAR PRO). The results were compared with USP specifications for pH levels [34].

### Packaging and labelling

Good Manufacturing Practice covers all aspects of manufacturing operations including packaging and labelling. Samples’ label on the bottles and other packaging materials were each examined for the recommended product information such as name, batch number, active pharmaceutical ingredients and amounts, expiry and manufacturing dates, storage conditions and precautions, name and physical address of the pharmaceutical manufacturer in compliance to GMP requirements [35].

### Ethical approval

This was obtained from Kenya Medical Research Institute, Scientific and Ethics Review Unit (KEMRI/SERU/CTMDR/012/3059). Co-trimoxazole is prescription only medicine hence authorization letter from Kenya Medical Research Institute allowing for purchase of the samples was presented.

## Results and discussion

### API content, pH and packaging

A total of 106 oral suspension samples that were of fifteen different brands of co-trimoxazole were analysed. Thirteen (86.7%) of these brands were of local origin (products labelled manufactured in Kenya) and the remaining two were from India and Egypt. Orwa [2008] [36] also reported many co-trimoxazole brands of local origin in a study that evaluated quality of some pharmaceutical products manufactured in Kenya. Retention status of these brands was confirmed from PPB website (https://products.pharmacyboardkenya.org) on 23^rd^ October 2019. It was noted that retention status of 40.0% (6 out of 15) of the oral suspension brands could not be confirmed as they were not found in the PPB retention register. Pharmacy and Poisons Board requires that once a pharmaceutical product has been approved and registered based on quality, safety and efficacy, the manufacturer or solo-distributor pays for annual retention for continued sale in the market [37].

Thirty-one samples of local origin had pH values that were above USP upper limit pH specifications of 6.5 (Table 2). Factors that could have resulted to pH failure were not established in this study. However, this could be attributed to poor quality assurance employed during manufacturing processes. The pH of liquid formulations indicates API stability and microbial quality of a product over time [25]. It is worrying that 31 out of 106 samples that were of local origin failed this requirement. To the contrary, Frimpong et al. [2018] [25] carried out a study on children formulation in Ghana where all five co-trimoxazole samples passed the pH and microbial load tests but failed API requirement. World Health Organization, recommends use of co-trimoxazole prophylaxis on infants born to HIV infected mothers from age 4–6 weeks, until cessation of breast feeding and exclusion of HIV infection [38]. Besides the API content, it is important that a drug also meets physicochemical parameters such as pH for assurance of quality, stability and effectiveness.

**Table 2:** Results for active pharmaceutical ingredient, pH and retention status.

Sample Code	Country of Origin	pH (5.0–6.5)	pH Remarks	Assay % TMP	Assay % SMZ	Retention Status	Remarks on API
**Control**	**Egypt**	**5.210**	**Passed**	**98.45(0.78)**	**100.7(1.45)**	Retained	**Passed**
KB072-1	Local	6.783	**Failed**	**98.9(1.08)**	**104.4 (1.11)**	Retained	**Passed**
EMS207-1	Local	6.735	**Failed**	**93.0(0.59)**	**91.1(0.67)**	Retained	**Passed**
KB057-13	Local	6.537	**Failed**	**98.2(1.61)**	**95.4(0.87)**	Retained	**Passed**
MKB111-5	Local	6.531	**Failed**	**97.8(1.33)**	**102.1(1.13)**	Retained	**Passed**
EBC061-1	Local	6.580	**Failed**	**92.9(0.34)**	**94.41(0.16)**	Retained	**Passed**
DGN37-2	Local	6.596	**Failed**	**96.5(1.42)**	**99.6(1.28)**	Retained	**Passed**
MT200-1	Local	6.661	**Failed**	**94.6(0.72)**	**95.9(0.37)**	Retained	**Passed**
KS175-4	Local	6.643	**Failed**	**96.7(0.35)**	**93.4(1.15)**	Retained	**Passed**
KB73-01	Local	6.579	**Failed**	**100.0(0.94)**	**104.8(1.98)**	Retained	**Passed**
RY276-1	Local	6.614	**Failed**	**93.5(0.41)**	**101.6(0.25)**	Retained	**Passed**
ST149-4	Local	6.596	**Failed**	**95.5(0.27)**	**99.8(0.98)**	Retained	**Passed**
WS124-2	Local	6.702	**Failed**	**93.0(0.88)**	**94.08(0.81)**	Retained	**Passed**
RA333-1	Local	6.502	**Failed**	**94.3(1.89)**	**97.5(1.48)**	Retained	**Passed**
KM131-4	Local	6.715	**Failed**	**92.9(1.65)**	**92.9(1.65)**	Retained	**Passed**
WS01-1	Local	6.603	**Failed**	**86.22(0.46)**	**90.46(1.17)**	Retained	**Failed**
EBN179-1	Local	6.775	**Failed**	**96.6(1.30)**	**101.5(0.81)**	Retained	**Passed**
KS261-2	Local	6.502	**Failed**	**93.1(0.83)**	**101.0(1.94)**	Retained	**Passed**
RY253-3	Local	6.697	**Failed**	**91.8(1.01)**	**97.6(1.23)**	Retained	**Passed**
RA191-5	Local	6.655	**Failed**	**92.6(1.74)**	**98.1(1.62)**	Retained	**Passed**
EBW098-2	Local	6.639	**Failed**	**91.5(0.56)**	**91.0(0.83)**	Retained	**Passed**
MT201-2	Local	6.625	**Failed**	**97.1(1.79)**	**103.1(1.88)**	Retained	**Passed**
ST159-10	local	6.732	**Failed**	**100.7(0.75)**	**103.6(1.82)**	Retained	**Passed**
EBW119-2	Local	6.600	**Failed**	**90.47(0.57)**	**96.65(0.55)**	Retained	**Passed**
WS133-3	Local	6.679	**Failed**	**102.58(1.15)**	**106.32(0.84)**	Retained	**Passed**
DGS039-1	Local	5.011	**Passed**	**103.2(0.27)**	**99.1(0.46)**	Missing	**Passed**
DGN042-2	Local	6.011	**Passed**	**85.6(0.47)**	**90.0(0.99)**	Missing	**Failed**
EBC188-1	Local	5.947	**Passed**	**91.3(0.99)**	**96.70(2.17)**	Missing	**Passed**
EBE272-6	Local	5.918	**Passed**	**93.41(0.82)**	**100.5(0.40)**	Missing	**Passed**
KM242-6	Local	5.950	**Passed**	**104.6(1.89)**	**97.0(0.19)**	Missing	**Passed**
EBS206-7	Local	5.987	**Passed**	**95.68(1.10)**	**99.14(1.05)**	Missing	**Passed**
WS114-1	Local	6.013	**Passed**	**95.9(1.39)**	**96.6(0.76)**	Missing	**Passed**
RY277-2	Local	5.982	**Passed**	**101.60(0.31)**	**108.7(0.96)**	Missing	**Passed**
MT136-2	Local	6.097	**Passed**	**95.3(0.41)**	**105.6 (1.57)**	Retained	**Passed**
KB021-1	Local	6.088	**Passed**	**92.2(1.05)**	**99.82(0.28)**	Retained	**Passed**
EMS192-1	Local	5.471	**Passed**	**98.1(0.23)**	**96.0(0.73)**	Retained	**Passed**
KM246-11	Local	5.287	**Passed**	**89.71(0.74)**	**96.4(0.11)**	Missing	**Failed**
MT218-1	Local	5.394	**Passed**	**89.7(0.84)**	**90.7(1.92)**	Missing	**Failed**
EBC058-1	Local	6.103	**Passed**	**93.1(0.34)**	**97.8(0.57)**	Retained	**Passed**
ST171-1	Local	6.146	**Passed**	**90.47(0.92)**	**95.89 (1.54)**	Retained	**Passed**
EBC108-1	Local	5.986	**Passed**	**89.2(1.34)**	**95.98(0.85)**	Retained	**Failed**
DGS401-3	Local	6.200	**Passed**	**95.9(0.22)**	**96.4(1.17)**	Retained	**Passed**
KB050-1	Local	6.186	**Passed**	**94.7 (0.61)**	**97.61 (1.51)**	Retained	**Passed**
EBW120-1	Local	6.121	**Passed**	**95.6(0.78)**	**99.6(0.48)**	Retained	**Passed**
RY321-1	Local	5.947	**Passed**	**95.8(1.36)**	**93.6(1.47)**	Retained	**Passed**
KS291-1	Local	6.090	**Passed**	**90.4(1.03)**	**96.2(0.98)**	Retained	**Passed**
LG007-2	Local	6.087	**Passed**	**93.8(1.31)**	**98.4(1.53)**	Retained	**Passed**
MKB109-2	Local	6.074	**Passed**	**95.5(0.29)**	**95.21(0.25)**	Retained	**Passed**
WS168-3	Local	6.168	**Passed**	**91.9(1.19)**	**97.3(0.97)**	Retained	**Passed**
EBW123-2	Local	6.404	**Passed**	**94.5(0.12)**	**92.4(0.80)**	Missing	**Passed**
KB033-1	Local	5.607	**Passed**	**82.63(1.63)**	**82.20(1.61)**	Missing	**Failed**
EBE311-1	Local	6.050	**Passed**	**101.7(1.22)**	**104.9(1.39)**	Missing	**Passed**
MT236-1	Local	6.065	**Passed**	**96.8(1.28)**	**102.7(1.63)**	Missing	**Passed**
EBN178-2	Local	5.904	**Passed**	**101.8(1.59)**	**93.8(1.56)**	Missing	**Passed**
EBE-181-1	Local	5.506	**Passed**	**99.6(1.66)**	**98.7(0.78)**	Missing	**Passed**
MT181-1	Egypt	5.878	**Passed**	**97.3(0.17)**	**99.0(1.81)**	Retained	**Passed**
KM231-1	Egypt	5.945	**Passed**	**98.4(1.85)**	**102.0(1.47)**	Retained	**Passed**
ST128-3	Egypt	5.809	**Passed**	**100.1(1.12)**	**97.1(1.24)**	Retained	**Passed**
EBE306-2	Egypt	5.931	**Passed**	**105.8(1.73)**	**107.8(1.19)**	Retained	**Passed**
EBW 96–1	Egypt	5.870	**Passed**	**102.5(0.44)**	**95.4(0.14)**	Retained	**Passed**
EBW96-1	Egypt	5.891	**Passed**	**102.5(0.44)**	**95.4(0.14)**	Retained	**Passed**
DGS35-2	Egypt	6.006	**Passed**	**99.7(1.40)**	**94.5(1.34)**	Retained	**Passed**
KB076-1	Local	5.331	**Passed**	**86.8(1.35)**	**95.65(1.56)**	Retained	**Failed**
MT136-4	Local	5.487	**Passed**	**100.4(1.76)**	**105.1(0.95)**	Retained	**Passed**
KB053-7	Local	5.356	**Passed**	**104.24(0.38)**	**113.4(0.73)**	Retained	**Failed**
RY276-2	Local	5.982	**Passed**	**92.90(0.72)**	**102.4 (0.98)**	Retained	**Passed**
DGN045-1	Local	6.125	**Passed**	**97.5(1.15)**	**101.9(1.46)**	Retained	**Passed**
DGN047-1	Local	6.046	**Passed**	**101.8(1.86)**	**101.7 (1.47)**	Retained	**Passed**
ST169-1	Local	6.051	**Passed**	**90.2(0.44)**	**97.3(0.98)**	Retained	**Passed**
KB050-3	Local	5.923	**Passed**	**102.1(0.37)**	**104.4(1.93)**	Retained	**Passed**
WS139-2	Local	6.009	**Passed**	**94.4(0.93)**	**96.5(0.31)**	Retained	**Passed**
EBE282-1	Local	5.965	**Passed**	**98.9(0.75)**	**104(0.58)**	Retained	**Passed**
EBC187-1	Local	5.966	**Passed**	**100.2(0.9)**	**100.8(0.96)**	Retained	**Passed**
RY315-1	Local	6.098	**Passed**	**99.1(0.96)**	**100.92(1.04)**	Retained	**Passed**
KS269-1	Local	6.017	**Passed**	**96.9(0.30)**	**100.7(0.57)**	Retained	**Passed**
EBW090-1	Local	6.109	**Passed**	**101.2(1.74)**	**99.0(0.94)**	Retained	**Passed**
MT257-1	Local	6.064	**Passed**	**93.1(0.08)**	**95.4(0.38)**	Retained	**Passed**
MK103-4	Local	5.984	**Passed**	**96.4(0.89)**	**102.5(0.82)**	Retained	**Passed**
WS143-1	Local	6.056	**Passed**	**100.8(0.28)**	**107.5(1.13)**	Retained	**Passed**
KB043-1	Local	6.094	**Passed**	**96.7(0.12)**	**103.9(1.05)**	Retained	**Passed**
EMS205-9	Local	6.073	**Passed**	**105.8(1.89)**	**99.93(1.93)**	Retained	**Passed**
DGS012-2	Local	5.072	**Passed**	**103.4(0.447)**	**109.3 (0.42)**	Retained	**Passed**
EBW095-3	Local	5.006	**Passed**	**91.5(1.49)**	**93.4(1.45)**	Retained	**Passed**
MK85-2	Local	5.589	**Passed**	**97.11(0.38)**	**94.5(0.05)**	Retained	**Passed**
RY289-2	Local	6.513	**Failed**	**99.8(1.48)**	**95.7(0.62)**	Missing	**Passed**
MT235-1	Local	6.311	**Passed**	**95.0(0.63)**	**99.8(0.25)**	Missing	**Passed**
EMS203-5	Local	6.440	**Passed**	**90.1(0.79)**	**90.51(0.93)**	Missing	**Passed**
EBN176-1	Local	5.949	**Passed**	**104.3(1.63)**	**102.5(0.76)**	Missing	**Passed**
EBC186-1	Local	6.650	**failed**	**93.5 (0.04)**	**99.8 (0.68)**	Missing	**Passed**

Figure in brackets is Relative Standard Deviation; Trimethoprim and Sulfamethoxazole USP limits: Not less than 90.0% and not more than 110.0% of label claim; 40mg Trimethoprim/ 200mg Sulfamethoxazole per 5 ml.

**Fig 1:**
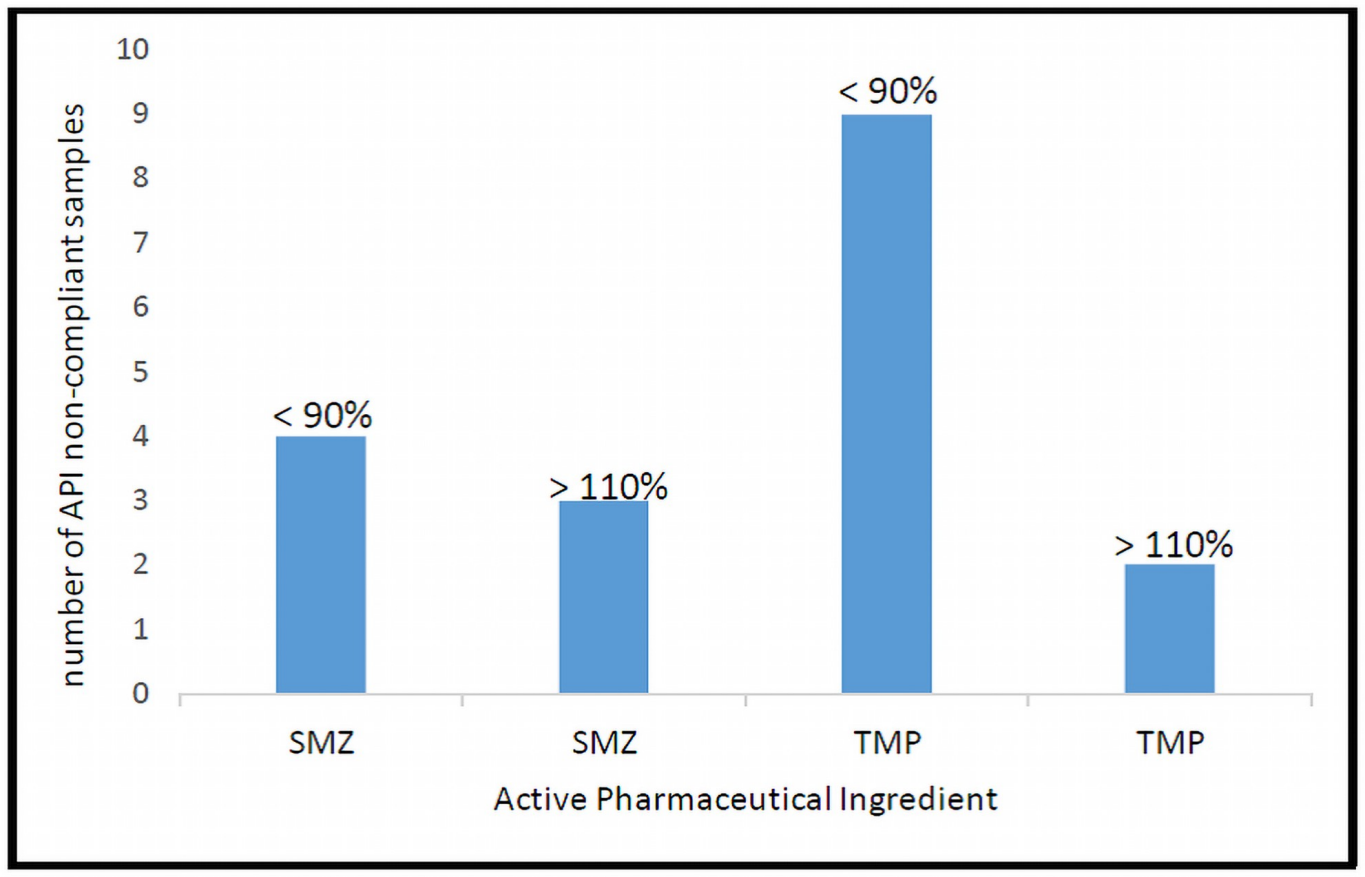
Distribution of API non-compliant samples in both lower and upper limits.

The active pharmaceutical ingredients, trimethoprim (TMP) and sulfamethoxazole (SMZ) were present in all the samples tested. However, for effective therapeutic efficacy, API content must be within the recommended pharmacopeia limits of 90–110% for both SMZ and TMP according to USP (2017). The samples content for SMZ ranged from 63.0%-116.8% while that of TMP was 82.6% - 115.9%. Of the 106 samples, 86.8% were compliant with USP specifications limits for both TMP and SMZ content as shown in Tables 2 and 3. Fourteen (14) samples failed to meet pharmacopeia API limits where 28.6% (4/14) were non-compliant for both TMP and SMZ, 21.4% (3/14) and 50.0% (7/14) for sulfamethoxazole and trimethoprim respectively. A major cause for non-compliance to required pharmacopeia limits was insufficient APIs, but cases of excess were also noted as shown in Fig 1. This observation could suggest poor adherence to good manufacturing practices and is further corroborated by intra-batch variation observed in Table 3. Excess API poses a risk of toxicity to the patient while insufficient API leads to a sub therapeutic dosage which could promote drug resistance [39]. Also, intra-batch variation was observed in samples collected from different locations (Table 3) such as EMS199-1 and KS259-1 that were non-compliant for both SMZ and TMP; EBW119-1 for SMZ and KM227-1 for TMP. Intra-batch variation is reported in other studies [11, 39] and could be partly attributed to poor adherence to good manufacturing practices [40].

**Table 3:** Results for samples with intra-batch variation: Active pharmaceutical ingredient, pH and retention status.

Sample Code	Country of Origin	pH (5.0–6.5)	pH Remark	Assay % TMP	Assay % SMZ	Retention Status	API remark
^a^EBE280-2	India	6.048	**Passed**	**103.1 (0.57)**	**107.7(0.73)**	**Retained**	**Passed**
^a^EBC182-3	India	6.104	**Passed**	**105.6(00.99)**	**106.8(0.57)**	**Retained**	**Passed**
^a^EBN214-1	India	5.886	**Passed**	**107.8(0.94)**	**110.4(0.29)**	**Retained**	**Passed**
^a^EMS199-1	India	6.073	**passed**	**115.5(1.74)**	**116.8(00.65)**	**Retained**	**Failed**
^a^EBW097-1	India	5.867	**Passed**	**105.8(1.31)**	**109.5(1.28)**	**Retained**	**Passed**
^a^WS133-1	India	5.501	**Passed**	**104.8 (1.19)**	**97.6(0.83)**	**Retained**	**Passed**
^a^RA300-1	India	5.781	**Passed**	**105.1(1.27)**	**109.7(1.48)**	**Retained**	**Passed**
^b^EBC182-4	Local	6.649	**Failed**	**92.7(1.50)**	**98.2(1.75)**	Retained	**Passed**
^**b**^EBW119-1	Local	6.651	**Failed**	**100.9(1.09)**	**63.01(1.06)**	Retained	**Failed**
^b^MT136-6	Local	6.859	**failed**	**98.3(0.24)**	**97.41(1.77)**	Retained	**Passed**
^c^MK086-3	Local	6.659	**Failed**	**93.9(0.87)**	**98.4(0.88)**	Retained	**Passed**
^c^KM227-1	Local	6.662	**Failed**	**86.87 (1.89)**	**98.5(1.22)**	Retained	**Failed**
^d^KM243-7	Local	6.064	**Passed**	**104.4(1.01)**	**109.9(0.40)**	Retained	**Passed**
^d^ST140-2	Local	6.082	**passed**	**115.91(0.64)**	**111.35(0.43)**	Retained	**Failed**
_e_EBE309-1	Local	5.699	**Passed**	**105.3(0.63)**	**108.6(1.94)**	Missing	**Passed**
^e^KS290-2	Local	5.756	**Passed**	**90.6(1.98)**	**89.98(1.69)**	Missing	**Failed**
^f^KS259-1	Egypt	5.869	**Passed**	**84.7(1.67)**	**87.85(1.61)**	Retained	**Failed**
^f^KM245-10	Egypt	5.864	**Passed**	**93.3(1.94)**	**97.8(1.85)**	Retained	**Passed**

Samples bearing same alphabet belongs to the same batch; Figure in brackets is Relative Standard Deviation

Ten of the samples that failed to comply with API requirements were of local origin while 4 were imports. Though this study could not relate quality of a brand to retention with PPB register, it was noted that four of the samples whose API limits were non-compliant missed out in the retention register. A study in Kenya by Orwa [2008] [36] corroborates the low API failure rate incidence where 9 out of 10 samples of co-trimoxazole suspension tested met British Pharmacopeia specification for API content.

Samples that did not meet pharmacopeia requirements were documented in 8 out of 17 Sub-counties. They were sampled from Embakasi and Kibra at 2.8%, Kasarani and Kamukunji at 1.9% each; Westlands, Dagoretti, Starehe and Mathare at 0.9% each. Of note, samples that failed pharmacopeia limits were collected across all the social economic setting. Hence, quality could not be directly associated with location of purchase in this study.

Previous studies have reported poor quality co-trimoxazole suspensions and tablets [4, 16, 20, 41]. In Nigeria, a study by Lawal et al. [2019] [41] reported that 7 out of 17 co-trimoxazole tablets fell outside of pharmacopeia limit and were the highest proportion of samples that failed out of 6 different antibiotics that were tested. Similarly, Frimpong et al. [2018] [25] in a study carried out in Ghana reported that 5 co-trimoxazole suspension samples tested failed the API requirements but passed pH and microbial load tests. Substandard medicines with either under or over API content threaten health, causing poor treatment outcome and prolonged illness and promoting development of drug resistance [42]. This emphasizes the need for manufacturers to ensure that drugs of acceptable quality reach the patient by adhering to good manufacturing practices.

**Fig 2:**
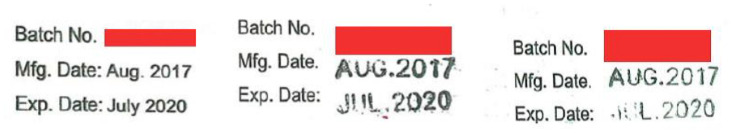
Labels of samples with same batch number but different text font and style.

**Fig 3:**
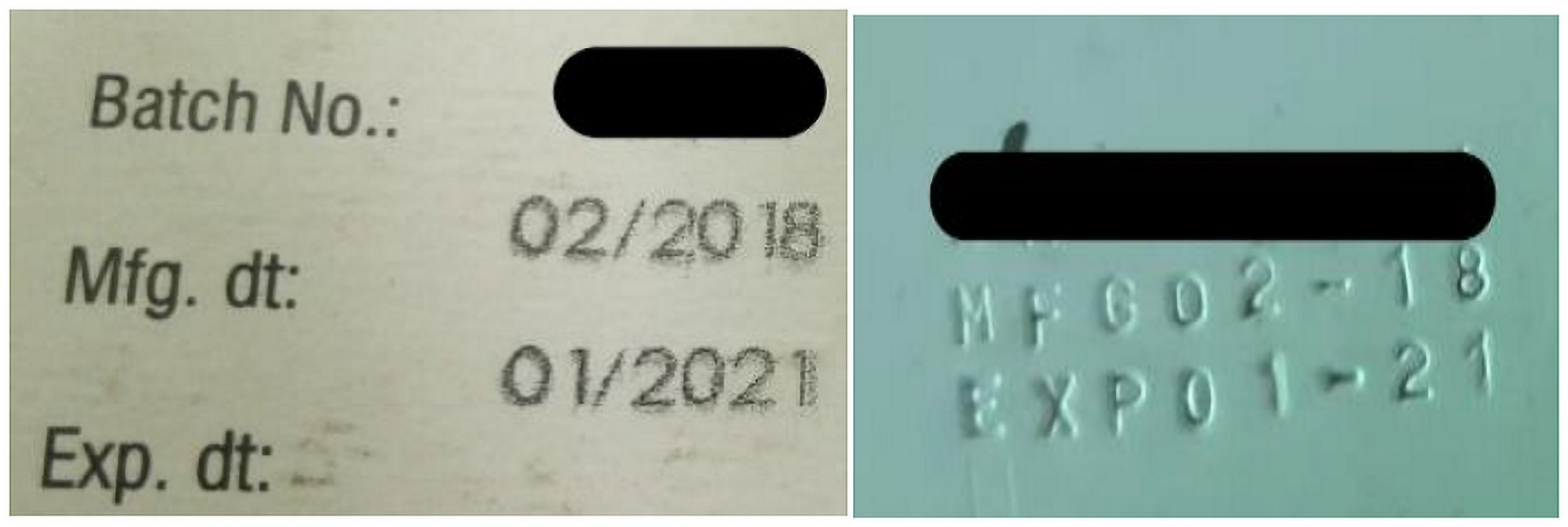
Labels of samples with same batch number of different printing style (Ink and engraved).

**Fig 4:**
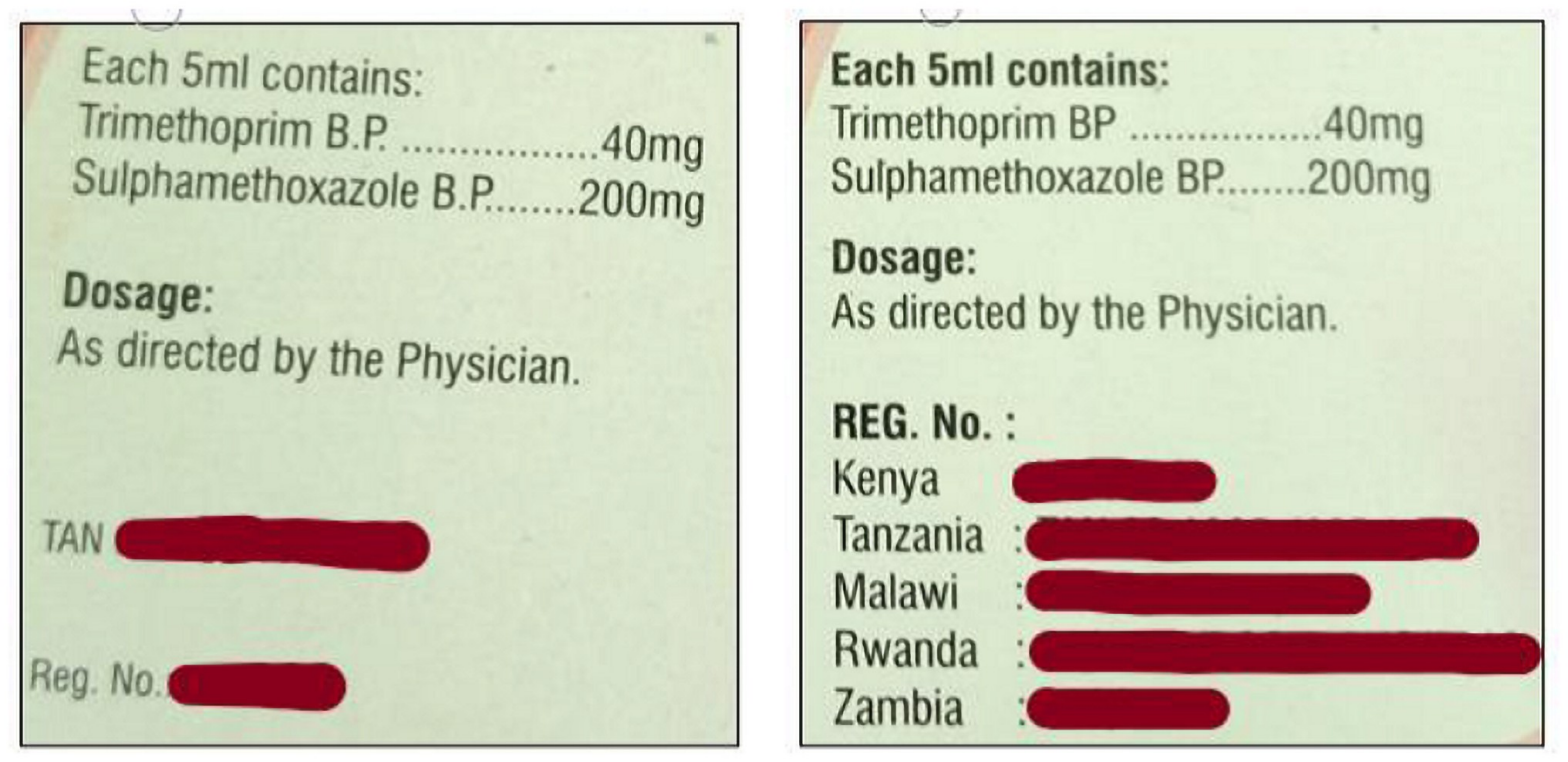
Samples with same batch number but different information on the product pack.

Wrong labelling is one of the main risks in pharmaceutical manufacturing as it could lead to patients receiving the wrong medicine [35]. Majority (89 out of 106) of the samples tested adhered to packaging and labelling requirements. However, labelling errors were noted where 6.7% of the samples had inconsistent printing of similar batch numbers, manufacture and expiry dates; 1% had missing bottle labels and 7.6% had poor labelling with batch numbers and expiry dates being totally blurred or unclear. Samples with labelling errors were of local origin with the exception of one. Finished product labels must conform to international requirements [35]. The information appearing on labels should be clear and legible. Of great concern was the existence of samples with same batch number but of different printing styles (font, size, ink and engraved print) as shown in Figs 2 and 3. WHO defines a batch as a quantity of pharmaceutical products processed in a single process or a series of processes that is homogeneous [43]. Samples EBC182-4, EBW119-1 and MT136-1 had the same batch number whose print was not similar in regard to size and font (Fig 2). All the 3 samples had their pH above the limits at 6.649, 6.51 and 6.859 for EBC182-4, EBW119-1 and MT136-1 respectively. It was also noted that the SMZ content for EBW119-1 was below pharmacopeia limits. These results suggest that the samples may not be a single batch despite sharing a batch number. This particular brand was also missing from the PPB retention list. However, this study did not directly associate the quality of the three samples to the retention status. Similarly, samples LG007-2 and WS168-3 shared a batch number printed with ink and the other engraved as shown in Fig 3. The samples also had different information printed on the packaging (Fig 4). It was observed that their pH values were within the same range (6.087 and 6.168) and API for trimethoprim and sulfamethoxazole were also within range (Table 1) an indication that the samples though bearing different printing styles and information on the packaging could be of the same batch. A recent study carried out in Democratic Republic of Congo by Schiavetti et al. [2018] [40] similarly reported poor labelling where samples were without license number, missing manufacturer details and generally poor printing quality.

### Study limitation

Results of this study are limited to analysis of API content using HPLC (Diode array -UV detector) and determination of pH values of co-trimoxazole oral suspension. Microbial load that could have provided complementary information on quality of the suspensions was not done due to insufficient research funds. The study focused on Nairobi County; hence the findings may not be extrapolated to other counties within Kenya.

## Conclusion

The study has demonstrated that majority of co-trimoxazole suspensions tested were compliant with API content (86.8%) and labelling requirements (84.0%). However, 29.2% failed to meet pH requirements which is a pointer to the quality and efficacy of the medicines over time. Therefore, there is need to analyse these medicines over time to ascertain their efficacy. Sixteen percent of the samples failed to adhere to the pharmacopeia packaging and labelling requirements [35]. This is evident of the need for strict regulation to guarantee compliance to good manufacturing practices by pharmaceutical manufacturers. Co-trimoxazole is an essential medicine that plays a major role as a prophylaxis in management of Human Immunodeficiency Virus [44]. Ensuring quality of such medicines contributes towards achieving the Universal Health Coverage that Kenya targets to achieve by the year 2030.

## Supporting information

S1 Appendix Co-trimoxazole drug purchase data sheet. (DOCX)

S1 Table Distribution of API non-compliant samples in both lower and upper limits. (DOCX)
