# Supplementary material for: Quality assessment of selected co-trimoxazole suspension brands marketed in Nairobi County, Kenya
Source: PLoS One. 2021 Sep 22;16(9):e0257625. doi: 10.1371/journal.pone.0257625 (PMC8457504; doi:10.1371/journal.pone.0257625)
Supplement: S1 Appendix — (DOCX) [file pone.0257625.s001.docx]

**S1 Appendix:** CO-TRIMOXAZOLE DRUG PURCHASE DATA SHEET

**Sub County: …………………………………….. Latitude: ………………………………………..**

**Ward: …………………………..................... Longitude: …………………………………….**

**Date: ………………………………………………… Attitude: ……………………………………….**

**Green Cross No: ……………………………**

| **Sample Code** | **Brand Name and strength** | **Batch Number** | **Country of origin** | **Manu. Company/**  **Distributor** | **Manu. Date** | **Expiry Date** | **Price**  **/dose/bottle** |
| --- | --- | --- | --- | --- | --- | --- | --- |
|  |  |  |  |  |  |  |  |
|  |  |  |  |  |  |  |  |
|  |  |  |  |  |  |  |  |
|  |  |  |  |  |  |  |  |
|  |  |  |  |  |  |  |  |
|  |  |  |  |  |  |  |  |
|  |  |  |  |  |  |  |  |
|  |  |  |  |  |  |  |  |
|  |  |  |  |  |  |  |  |

DATA COLLECTORS: 1. …………………………………………… 2. ……………………………………………
