# Supplementary material for: Quality assessment of selected co-trimoxazole suspension brands marketed in Nairobi County, Kenya
Source: PLoS One. 2021 Sep 22;16(9):e0257625. doi: 10.1371/journal.pone.0257625 (PMC8457504; doi:10.1371/journal.pone.0257625)
Supplement: S1 Table — (DOCX) [file pone.0257625.s002.docx]

S1 Table**: Distribution of API non-compliant samples in both lower and upper limits**

| **Limit** | **API** | **Number of non-compliant samples** |
| --- | --- | --- |
| <90% | SMZ | 4 |
| >110% | SMZ | 3 |
| <90% | TMP | 9 |
| >110% | TMP | 2 |
